# The association between dietary branched-chain amino acids and the risk of cardiovascular diseases in Chinese patients with type 2 diabetes: A hospital-based case–control study

**DOI:** 10.3389/fnut.2022.999189

**Published:** 2022-10-14

**Authors:** Lu Zheng, Jun Cai, Yong-hui Feng, Xin Su, Shi-yun Chen, Jia-zi Liu, Wan-lin Li, Rui-qing Ouyang, Jun-rong Ma, Chen Cheng, Ying-jun Mu, Shi-wen Zhang, Kai-yin He, Fang-fang Zeng, Yan-bin Ye

**Affiliations:** ^1^Department of Public Health and Preventive Medicine, School of Medicine, Jinan University, Guangzhou, China; ^2^Department of Clinical Nutrition, The First Affiliated Hospital, Jinan University, Guangzhou, China; ^3^Department of Clinical Nutrition, The First Affiliated Hospital of Sun Yat-sen University, Guangzhou, China

**Keywords:** branched-chain amino acids, BCAA, isoleucine, leucine, valine, cardiovascular diseases, type 2 diabetes

## Abstract

**Background:**

Previous studies showed conflicting evidence on the association between the intake of dietary branched-chain amino acid (BCAA) and the risk of cardiovascular disease (CVD). However, this relationship has not been studied in patients with type 2 diabetes. Therefore, we evaluated the effects of total and individual dietary BCAA (leucine, isoleucine, and valine) intake on CVD risk among individuals with type 2 diabetes in China.

**Materials and methods:**

A total of 419 patients with type 2 diabetes who have been diagnosed with CVD (within 2 weeks) were recruited between March 2013 and September 2015 in China. Cases with CVD were 1:1 matched to controls with type 2 diabetes but without CVD by age (±5 years) and sex. A validated 79-item semiquantitative food frequency questionnaire (FFQ) was administered to assess the participants' dietary data. Total dietary BCAA per individual was the summation of the daily intake of isoleucine, leucine, and valine. OR and corresponding CIs were computed by conditional logistic regression models adjusted for potential confounders.

**Results:**

Median values of the daily intake of total BCAA were 11.87 g, with an interquartile range of 10.46–13.15 g for cases, and 12.47 g, with an interquartile range of 11.08–13.79 g for controls (*P* = 0.001). Dietary BCAA was inversely related to CVD risk after multivariable adjustment (OR _Q4−*Q*1_ = 0.23, 95%CI = 0.10, 0.51, *P* trend <0.001 for total BCAA; OR _Q4−*Q*1_ = 0.20, 95%CI = 0.07, 0.53, *P* trend = 0.001 for leucine). For each 1-S.D. increase in total dietary BCAA, leucine or valine intake was associated with 54% (95%CI = 29%, 70%, *P* = 0.001), 64% (95%CI = 29%, 82%, *P* = 0.003), or 54% (95%CI = 1%, 79%, *P* = 0.049) decrease in the risk of CVD, respectively. Whole grains, starchy vegetables, mushrooms, fruit, eggs, and dairy and dairy product-derived BCAA were found to attenuate CVD risk (*P* ranged: = 0.002–0.027).

**Conclusion:**

Higher BCAA intake, in particular leucine and valine, might be associated with a lower risk of CVD.

## Introduction

Type 2 diabetes is one of the risk factors for cardiovascular diseases (CVD) (1). The prevalence of diabetes among Chinese adults increased from 10.9% in 2013 to 12.4% in 2018 (2). Participants who shifted from impaired fasting glucose to diabetes had 61% increased risk for CVD and a 75% increased risk for congenital heart disease (CHD) (3). Similarly, the hazard ratio among the participants with insulin resistance, which later becomes type 2 diabetes, and the risk of CVD and CHD were 3.68 and 2.76, respectively (3). Thus, good management of type 2 diabetes in terms of lifestyle factor is necessary for preventing CVD in patients with type 2 diabetes.

Diets are considered as one of several fundamental factors in preventing or developing many chronic non-communicable diseases, including type 2 diabetes and CVD. Protein intake may play important role in the development of type 2 diabetes and possibly cardiovascular diseases (4). Previous studies from systematic reviews and meta-analyses indicated that the consumption of total protein and animal protein, including red meat and processed meat, was positively associated, while the consumption of plant protein, such as soy, was negatively associated with the risk of type 2 diabetes and CVD mortality (5, 6).

Branched-chain amino acids (BCAAs) such as leucine (Leu), isoleucine (Ile), and valine (Val) are exogenous essential amino acids derived from animal and/or plant protein-containing foods rather than endogenously synthesized (7). It was found that the BCAA diet had significant mediating effects on protein synthesis, glucose homeostasis, and obesity prevention (8). A systematic review and meta-analysis reported that while adherence to dietary BCAA, the risk of type 2 diabetes and obesity was increased by 1.32 and 0.62, respectively (9). Increased ingestion of dietary BCAA promotes thrombosis formation (10). In addition, adverse metabolic effects mediated by isoleucine and valine were reported in rodents (11). The actual benefit to metabolic health from dietary BCAA was observed during the restriction of dietary BCAA in mice (11). Circulating BCAA was positively associated with incident cardiovascular disease (12). The relationships between diets and the pathogenesis of diabetes coinciding with CVD might be caused by the metabolism of BCAA, which could be observed as an increase in circulating BCAA levels (13). The higher chance of being CVD in patients with type 2 diabetes could possibly be attributed to the increase in plasma BCAA that activate the mammalian target of rapamycin (MTOR) signaling pathway (14, 15). Higher intake of BCAA and the corresponding components have been suggested to be associated with lower triglyceride levels, blood pressure, obesity risk, and CVD risk or mortality; however, null associations were observed by other studies (Supplementary Tables 1, 2).

To our knowledge, no study has evaluated the effects of BCAA on CVD risk among type 2 diabetes subjects. Therefore, to better understand the association between dietary BCAA intake and CVD risk in type 2 diabetes subjects, we aimed to investigate the association between dietary BCAA intake and CVD risk in Chinese patients with type 2 diabetes.

## Materials and methods

### Study design

We performed a 1:1 matched hospital-based case–control study [one case with type 2 diabetes and a CVD diagnosis within 2 weeks was matched for age (±5 years) and sex with 1 control with type 2 diabetes only] at the Endocrinology Department, the Neurology Department, and the Cardiology Department of the First Affiliated Hospital of Sun Yat-sen University, Guangdong, China, between 2013 and 2015.

The study protocol, including other study-related documents, adhered to the Declaration of Helsinki guidelines and was approved by the Ethics Committee of the First Affiliated Hospital of Sun Yat-sen University [No. (2017)019]. All participants were asked to sign informed consent forms before any epidemiological data, and biological specimens were collected. We followed the reporting guidelines of the Strengthening the Reporting of Observational Studies in Epidemiology (STROBE) statement for observational studies (16).

### Study population

All patients aged between 30 and 85 years admitted to the Endocrinology Department, the Neurology Department, and the Cardiology Department of the First Affiliated Hospital of Sun Yat-sen University, between 2013 and 2015 were considered for inclusion in the study.

### Inclusion criteria

Patients who were either natives of Guangdong or had resided in Guangdong for at least 5 years and had a history of at least 2 years of type 2 diabetes were enrolled. Only those who were diagnosed with CVD within 2 weeks after the date of the type 2 diabetes diagnosis were considered cases. The controls recruited corresponded to the cases who were patients with type 2 diabetes, had never been self-reported or diagnosed with CVD, exhibited no symptoms of cardiac involvement, had normal EKG levels, and had negative exercise tests.

### Exclusion criteria

Patients who had confirmed type 1 diabetes or gestational diabetes mellitus (GDM) and a history of cancer, hepatic disease, renal disease, autoimmune disorders, diabetic retinopathy, and congenital heart disease were first excluded. Then, patients who were physically disabled, had a disturbance of consciousness, had significant changes in their dietary habits or routine activities over the previous year, had an incomplete dietary assessment (≥10% missing values), had an implausible intake of total daily energy (<700 or >4,200 kcal per day for males, <500 or >3,500 kcal per day for females), or refused to participate in the study were also excluded.

### Ascertainment of diseases

We ascertained individuals as having type 2 diabetes according to the American Diabetes Association. Type 2 diabetes is defined as a fasting plasma glucose level ≥7.0 mmol L^−1^, a 2-h plasma glucose level ≥11.1 mmol L^−1^, or an A1C level ≥6.5% (17). The assessments of CVD were described in a previous study (18). Briefly, CVD referred to non-fatal acute myocardial infarction, hospitalization for unstable angina, and non-fatal stroke. The China Society of Cardiology of the Chinese Medical Association has provided diagnostic criteria for non-fatal myocardial infarction and (19) and unstable angina (20), comprising typical symptoms, elevated cardiac enzyme levels, and electrocardiographic findings.

### Data collection

#### Dietary assessment

A validated and reproducible 79-item semiquantitative food questionnaire (FFQ) was used to collect dietary information from each case and control (21). All cases completed dietary questionnaires during the 12 months prior to the diagnosis of a CVD. Both cases and controls were assessed for dietary intake *via* a face-to-face interview by a well-trained dietitian. For each food item, participants were asked to report on a frequency range of never, per day, per week, per month, and per year and to report the quantity of the average consumption of each food item. A photobook with different food portion sizes was provided to more precisely estimate the quantity of food intake.

All frequencies were converted to daily intake by dividing each possible frequency by 7 (per week), 30 (per month), and 365 (per year), and the portion sizes of each food item were calculated into grams per day. Energy, isoleucine, leucine, valine, and protein intake were calculated according to the China Food Composition Tables 2009, which includes the nutrient portion and energy of each food item (22). Total BCAA intake was obtained by the summation of isoleucine, leucine, and valine values in 100 g of a food item for each participant. Due to the non-normality of nutrients, the nutrient density method was used to normalize all nutrient data. The nutrient density model is a traditional approach used in nutritional epidemiological studies to adjust for the effect of total caloric intake (23). The nutrient residual (energy-adjusted) model was applied in this study by conducting a regression analysis of the nutrient intakes of individuals based on their total caloric intakes as previously described (24). The residual model was expressed as energy-adjusted intake = a + b, where a = residual of subjects in the regression model with nutrient intake as the dependent variable and total energy intake as the independent variable, and b = the expected nutrient intake for a person with a mean energy intake. To further analyze whether dietary BCAA obtained from different sources would interfere with the risk of CVD, we further categorized different food items into 16 food groups (e.g., whole grains, refined grains, tubers, starchy vegetables, non-starchy vegetables, legumes, beans, soy and soy products, fruit, unprocessed meat, processed meat, poultry, fish and seafood, dairy and dairy products, and eggs) based on their common nutritional values (Table 1).

**Table 1:** Example of food items constituting the 16 food groups.

**Foods or food groups**	**Food items**
Whole grains	Whole grain breakfast cereal, cooked oatmeal, wholewheat bread, purple rice, brown rice, other grains, bran
Refined grains	Refined grain breakfast cereal, white rice, white bread or bun, biscuits, waffle, crackers, pasta
Tubers	French fried, baked or mashed potatoes, potatoes, sweet potatoes, cassava, arrowroot, yam, taro, tapioca,
Mushrooms	Shiitake and relatives, oyster mushroom, wood ear mushroom, button mushroom, needle mushroom
Starchy vegetables	Carrot, pumpkin, broccoli, lotus root, wax gourd, towel gourd, other gourds
Non-starchy vegetables	Tomatoes, lettuce and mixed salad greens, lettuce salad, kale, spinach, other dark green leafy vegetables, green or red pepper or chili
Legumes	String beans, beans or lentils, peas or lima beans
Beans	Green beans, black beans, kidney beans, pinto beans
Soy and soy products	Soybean, tofu, soymilk
Fruit	Apple or juice, guava, orange or juice, grapefruit or juice, other non-sterile fruit juice, berries, mangoes, papaya, melons, kiwi, banana, water melon
Unprocessed meat	All red meat or offal such as pork, beef, lamb, liver, heart, kidney, spleen, tripe
Processed meat	Hot dog, sausage, bacon, ham, or smoked meat
Poultry	Chicken with skin, chicken without skin, goose, duck
Fish and seafood	Canned meat fish, sardine or salmon or anchovies or bluefish or trout or other fish, shrimp or prawn or lobster, scallops or oysters or mussels or clams, squid, octopus, sea crab
Dairy and dairy products	Whole milk or whole milk powder, skim low fat milk or skim low fat milk powder, sour cream, sherbet, ice cream, cheese, cottage, yogurt
Eggs	Eggs

#### Other covariates

Demographic information such as sex, education level (primary school or less, secondary/high school, college/university, or above), lifestyle habits (e.g., tobacco smoking, alcohol drinking status, tea drinking, and physical activity), history of chronic diseases, and medicine use (e.g., hypertension, dyslipidemia, insulin use, and oral hypoglycemic use) were collected for both cases and controls using a structured questionnaire *via* a face-to-face interview by a well-trained dietitian. Physical activity was evaluated by the Chinese version of the IPAQ (25), in which the metabolic equivalents-hour per week (MET-h/week) was computed to determine the activity level for each individual.

Anthropometric data, including weight (kg) and height (m), were ascertained using standard procedures and measuring equipment by trained personnel. We calculated BMI for each individual by dividing each individual weight in kilograms by their height in meters squared. We defined smokers or alcohol drinkers as smoking at least one cigarette per day or drinking alcohol once a week continuously for at least 6 months. Tea drinkers were people who drank tea at least twice a week. Individuals with a mean systolic pressure (SBP) ≥140 mmHg and/or a diastolic blood pressure (DBP) ≥90 mmHg and/or self-reported antihypertensive therapy were classified as hypertensive.

Dyslipidemia was determined according to the China Atherosclerosis Society guidelines and was defined as a cholesterol level of ≥200 mg/dl, a low-density lipoprotein cholesterol (LDL-c) level of ≥130 mg/dl, a high-density lipoprotein cholesterol (HDL-c) level of <40 mg/dl, triglyceride (TG) level of ≥160 mg/dl, or lipid-lowering drug use (26).

### Statistical analyses

The normality of continuous variables was checked by the Kolmogorov–Smirnov test before running any statistical analysis. Demographic data among participants were summarized by descriptive statistics. Continuous normally distributed variables are expressed as the mean ± S.D., and skewed distributions are expressed as medians and interquartile ranges. Categorical variables are expressed as frequencies or percentages. For group comparisons, a paired *t*-test was applied for continuous variables with a normal distribution, whereas the Wilcoxon signed-rank test was used for non-normally distributed variables. The Pearson chi-square or Fisher's exact test was used for the categorical variables. The correlation coefficients between the intake levels of each BCAA and CVD risk factors, as well as protein intake adherence to food groups, were determined by Spearman's correlation coefficient. The total energy intake was adjusted for each food item using the residual method (23).

To determine the risk of CVD, total and individual BCAA intake were analyzed as both categorical and per S.D. increments. We grouped energy-adjusted intakes of total or individual BCAA into quartiles (Q_1_-Q_4_) based on control subjects by sex, and the sex-specific cutoffs were then applied to the cases. The lowest quartile (Q_1_) served as the reference group. Odds ratios (ORs) and 95% confidence intervals (CIs) were estimated using conditional logistic regression with different models. The first model was adjusted for age and sex. The second model was performed based on Model 1 and further adjusted for BMI (kg/m^2^), hypertension (SBP ≥ 190 mmHg and/or DBP ≥ 90 mmHg), physical activity (MET-h/day), antihypertensive drug use (yes/no), smoking status (yes/no), drinking status (yes/no), tea drinking (yes/no), and total energy intake (kcal/d). Last, a multivariable model was adopted to fully adjust for the second model and daily nutrient intake (g/d), including saturated fatty acids, total dietary fibers, and total dietary aromatic amino acids (AAAs). *P* < 0.05 were considered significant. All statistical tests were two-sided using SPSS version 26.0 (IBM Corp, Armonk, NY).

## Results

The mean ± standard deviation for age was 62.08 ± 9.67 years for cases and 62.06 ± 9.55 years for controls. This study presented a mean BMI of 24.08 kg/m^2^, in which the BMI of cases was substantially higher than that of controls (*P* = 0.011). Our cases had higher systolic blood pressure, but were less likely to drink tea and had less daily physical activity (MET-h/d). Importantly, with respect to the controls, the cases had higher TG levels (*P* = 0.005) but lower total cholesterol (TC) levels (*P* = 0.037) (Table 2).

**Table 2:** Characteristics included cases and controls.

**Characteristics**	**Cases (*****n*** = **419)**		**Controls (*****n*** = **419)**		***P***
	**Mean/n**	**S.D./%**	**Mean/n**	**S.D./%**	
Age (years)	62.08	9.67	62.06	9.55	0.926
BMI (kg/m^2^)	24.36	3.23	23.80	3.46	0.011
WHR (male ≥ 0.9, female ≥ 0.85), *n* (%)	358	85.4	341	81.4	0.114
Blood pressure (mmHg)					
Systolic	136.77	21.38	130.20	17.46	<0.001
Diastolic	79.05	11.89	77.33	10.87	0.157
Education level, *n* (%)					0.001
<Middle school	163	39.0	148	35.4	
Middle/High school	131	31.3	101	24.2	
≥College	119	28.5	168	40.2	
Current smokers^†^, *n* (%)	131	31.4	115	27.4	0.208
Alcohol drinkers^‡^, *n* (%)	53	12.7	56	13.4	0.758
Tea drinkers^§^, *n* (%)	188	45.0	220	52.5	0.029
Antidiabetic medication therapy, *n* (%)	379	91.3	408	97.8	<0.001
Antihypertension drug use, *n* (%)	283	68.7	162	39.1	<0.001
Family history of diabetes, *n* (%)	48	11.5	135	32.2	<0.001
Family history of hypertension, *n* (%)	63	15.0	101	24.1	<0.001
Physical activity (MET-h/d)	25.64	3.86	26.07	3.93	0.009
Plasma glucose (mmol/L)^||^					
FPG	7.30	2.73	7.80	3.83	0.711
2hPG	11.35	5.53	11.20	6.43	0.789
HbA1C (%)	7.50	2.43	9.10	3.43	<0.001
Plasma lipids (mmol/L)^||^					
HDL	0.90	0.30	1.10	0.40	0.086
LDL	2.80	1.00	3.05	1.20	0.252
TG	1.60	1.30	1.40	1.00	0.005
TC	4.30	1.40	4.70	1.63	0.037

Test for differences of cases and controls using a paired t-test for normally distributed continuous variables; Wilcoxon signed-ranks test for skewed distributed continuous variables; Pearson's chi-square test or Fisher's exact test for categorical variables; *P* < 0.05 indicates significant difference.

^||^Values are presented as median (IQR).

^†^Current smokers refer to participants who had been smoking at least one cigarette per day.

^‡^Alcohol drinkers refer to participants who had been smoking drinking alcohol once a week continuously for at least 6 months.

^§^Tea drinkers refer to participants who had been drinking tea at least twice a week.

Daily dietary nutrients and food group intake among cases and controls are shown in Tables 3, 4, respectively. Cases had lower total dietary BCAA intake than controls (median = 11.87, IQR = 2.68 g/d vs. median = 12.47, IQR = 2.71 g/d, *P* = 0.001), of which the consumption levels of dietary isoleucine, leucine, and valine were found to be significantly lower in cases (all *P* < 0.05). The level of total protein intake was lower for cases. For cases, the total BCAA from animal-based foods was higher than those from plant-based foods (*P* = 0.001). In addition, the daily intake of refined grain and processed meat was significantly higher in cases, while the daily intake of total energy, whole grains, mushrooms, vegetables, legumes, soy and soy products, fruit, dairy and dairy products, and eggs was obviously decreased (all *P* < 0.05).

**Table 3:** Daily dietary nutrients and food group intake after energy-adjusted among 419 pairs of cases and controls.

**Characteristics**	**Cases (*****n*** = **419)**		**Controls (*****n*** = **419)**		***P***
	**Median**	**IQR**	**Median**	**IQR**	
Total energy (kcal/d)	1,469.67	615.43	1,554.27	629.45	0.031
Total dietary fibers (g/d)	10.82	5.23	13.46	7.98	<0.001
Total carbohydrate (g/d)	212.11	37.20	215.23	36.35	0.625
Total fat (g/d)	131.10	109.41	153.63	151.65	0.003
Saturated fatty acids (g/d)	13.96	5.35	14.74	4.50	0.040
Total protein (g/d)	82.27	27.18	96.44	56.68	<0.001
Plant-based protein (g/d)	31.63	16.11	45.56	48.83	<0.001
Animal-based protein (g/d)	34.93	14.79	35.94	14.92	0.282
Total dietary BCAA (g/d)	11.87	2.68	12.47	2.71	0.001
Plant-based BCAA (g/d)	3.98	2.37	4.35	2.65	0.001
Animal-based BCAA (g/d)	6.10	2.48	6.31	2.68	0.171
Dietary isoleucine (g/d)	2.84	0.64	2.92	0.63	0.009
Dietary leucine (g/d)	5.63	1.13	5.87	1.17	<0.001
Dietary valine (g/d)	3.45	0.71	3.52	0.65	0.012

Plant-based protein included protein from vegetables (starchy vegetables and non-starchy vegetables), legumes, soy, grains (whole grains and refined grains), mushrooms, beans, tubers, and fruits.

Animal-based protein included protein from unprocessed meat, poultry, fish and seafood, eggs, dairy and dairy products, and processed meat.

Test for differences of cases and controls using the Wilcoxon signed-ranks test; *P* < 0.05 indicates significant difference.

**Table 4:** Distribution of food group intake after energy-adjusted among 419 cases and 419 controls.

**Characteristics**	**Cases (*****n*** = **419)**		**Controls (*****n*** = **419)**		***P***
**(g per day)**	**Median**	**IQR**	**Median**	**IQR**	
Whole grains	0.13	3.70	1.95	12.00	<0.001
Refined grains	521.87	194.72	493.03	157.77	0.004
Tubers	19.62	22.71	24.07	26.67	0.129
Mushrooms	26.53	38.37	33.19	56.78	0.003
Starchy vegetables	144.38	72.66	163.49	77.59	<0.001
Non-starchy vegetables	69.75	43.92	75.02	44.23	0.008
Legumes	11.18	18.49	13.57	18.76	0.040
Beans	23.24	30.07	24.88	30.84	0.329
Soy and soy products	14.57	30.64	21.38	44.92	<0.001
Fruit	24.66	40.48	40.79	52.63	<0.001
Unprocessed meat	99.56	49.51	92.82	54.47	0.143
Processed meat	1.07	2.29	0.74	1.53	0.001
Poultry	20.18	22.91	21.74	22.93	0.434
Fish and seafood	48.26	39.71	48.46	37.95	0.171
Dairy and dairy products	0.12	19.03	4.45	104.24	<0.001
Egg	22.96	17.12	26.11	26.29	<0.001

Test for differences of cases and controls using the Wilcoxon signed-rank test; *P* < 0.05 indicates significant difference.

The results revealed that the consumption levels of dietary isoleucine, leucine, and valine, as well as total dietary BCAA, were positively correlated with the intake amount of soy and soy products, vegetables, mushrooms, eggs, dairy and dairy products, poultry, red meat, and fish and seafood (all *P* < 0.05) (Table 5). Among all food groups, the intake level of total dietary BCAA was more strongly positively correlated with the intake of mushrooms (*r*_s_ = 0.464, *P* < 0.001), followed by the intake of fish and seafood, eggs, and soy and soy products. In contrast, the intake of starchy vegetables, poultry, red meat, dairy and dairy products, legumes, non-starchy vegetables, and whole grains had weaker positive relationships. We found that consuming more refined grains would lower amount of total BCAA (*r*_s_ = −0.291, *P* < 0.001) (Table 5).

**Table 5:** Spearman's correlation coefficients between dietary BCAA and protein intake adherence to food groups among 838 participants.

**Food groups (g/d)**	**Isoleucine (g/d)**	***P***	**Leucine (g/d)**	***P***	**Valine (g/d)**	***P***	**Overall BCAA (g/d)**	***P***
Whole grains	0.776	0.435	−0.003	0.930	−0.038	0.283	0.115	0.001
Refined grains	−0.261	<0.001	−0.215	<0.001	−0.137	<0.001	−0.291	<0.001
Soy and soy products	0.180	<0.001	0.159	<0.001	0.149	<0.001	0.231	<0.001
Non-starchy vegetables	0.111	0.002	0.092	0.008	0.111	0.002	0.127	<0.001
Starchy vegetables	0.138	<0.001	0.111	0.001	0.110	0.002	0.185	<0.001
Legumes	0.101	0.004	0.065	0.064	0.990	0.089	0.165	<0.001
Beans	0.087	0.013	0.056	0.108	0.010	0.772	0.088	0.012
Tubers	0.035	0.314	0.023	0.521	0.020	0.564	0.093	0.008
Mushrooms	0.184	<0.001	0.139	<0.001	0.147	<0.001	0.464	<0.001
Fruit	0.025	0.471	0.022	0.535	0.014	0.680	0.081	0.021
Eggs	0.217	<0.001	0.192	<0.001	0.214	<0.001	0.242	<0.001
Dairy and dairy products	0.118	0.001	0.134	<0.001	0.114	0.001	0.172	<0.001
Poultry	0.210	<0.001	0.223	<0.001	0.226	<0.001	0.180	<0.001
Processed meat	0.028	0.419	0.040	0.256	0.042	0.229	−0.015	0.676
Red meat	0.331	<0.001	0.369	<0.001	0.356	<0.001	0.177	<0.001
Fish and seafood	0.430	<0.001	0.397	<0.001	0.434	<0.001	0.367	<0.001

All values were adjusted for age, sex, BMI (kg/m^2^), hypertension (SBP ≥190 mmHg and/or DBP ≥90 mmHg), physical activity (MET-h/day), antihypertension drug use (yes/no), smoking status (yes/no), drinking status (yes/no), tea drinking (yes/no), total energy intake (kcal/d), and daily nutrients intake (g/d), including saturated fatty acids, total dietary fibers, and total dietary aromatic amino acids (AAAs).

*P* < 0.05 indicates significant difference.

The ORs, 95% Cis, and *P*-values for CVD risk according to levels of dietary BCAA intake are given in Table 6. Participants whose total dietary BCAA was in the highest quartile were 0.46 times less likely to have CVD than those in the lowest quartile after adjustment for age and sex (Model 1). When further adjustment was conducted for all possible covariances, there was a 0.23-fold risk of CVD for patients with type 2 diabetes in the highest quartile of total dietary BCAA (OR = 0.23, 95% CI = 0.10, 0.51, *P* trend ≤ 0.001, Model 3). When each specific BCAA was considered, the fully adjusted model (Model 3) revealed that the highest quartile of dietary isoleucine and leucine intake decreased the risk of CVD was 0.20 (95% CI = 0.07, 0.53, *P* = 0.001), while the consumption of isoleucine and valine showed no significant association, compared to the reference quartile. Similarly, significant negative associations between dietary leucine, valine, and total BCAA intake per S.D. and CVD risk in the fully adjusted model were simultaneously observed.

**Table 6:** Odd ratios and corresponding 95% confidence intervals according to dietary BCAA intake and the risk of CVD in type 2 diabetes individuals.

	**Quartile of BCAA intake (OR, 95%CI)**				***P* trend**	**BCAA intake per 1-S.D. (OR, 95%CI)**	***P***
	**Q1**	**Q2**	**Q3**	**Q4**			
**Isoleucine**							
*N* (case/control)	116/93	115/95	95/115	93/116			
Median (g/d)	2.34	2.74	3.02	3.42			
Model 1	1	0.99 (0.67, 1.46)	0.67 (0.45, 0.99)	0.64 (0.44, 0.95)	0.008	0.86 (0.75, 0.99)	0.031
Model 2	1	1.03 (0.66, 1.62)	0.71 (0.45, 1.12)	0.77 (0.49, 1.20)	0.125	0.90 (0.77, 1.07)	0.230
Model 3	1	0.79 (0.45, 1.39)	0.46 (0.22, 0.96)	0.45 (0.17, 1.19)	0.062	0.58 (0.26, 1.27)	0.174
**Leucine**							
N (case/control)	118/91	117/93	98/112	86/123			
Median (g/d)	4.74	5.48	6.00	6.71			
Model 1	1	0.96 (0.66, 1.40)	0.67 (0.45, 1.00)	0.54 (0.37, 0.81)	0.001	0.82 (0.72, 0.95)	0.006
Model 2	1	1.03 (0.66, 1.61)	0.75 (0.47, 1.20)	0.65 (0.41, 1.02)	0.026	0.88 (0.75, 1.04)	0.123
Model 3	1	0.62 (0.35, 1.10)	0.34 (0.16, 0.71)	0.20 (0.07, 0.53)	0.001	0.36 (0.18, 0.71)	0.003
**Valine**							
*N* (case/control)	117/92	106/104	101/109	95/114			
Median (g/d)	2.90	3.32	3.63	4.05			
Model 1	1	0.81 (0.55, 1.20)	0.75 (0.51, 1.10)	0.67 (0.45, 0.98)	0.037	0.88 (0.76, 1.01)	0.065
Model 2	1	0.79 (0.50, 1.26)	0.70 (0.44, 1.10)	0.74 (0.48, 1.16)	0.170	0.91 (0.78, 1.08)	0.275
Model 3	1	0.59 (0.33, 1.05)	0.44 (0.22, 1.00)	0.38 (0.14, 1.02)	0.113	0.46 (0.21, 0.99)	0.049
**Total BCAA**							
*N* (case/control)	123/86	112/98	102/108	82/127			
Median (g/d)	9.61	11.53	12.74	14.65			
Model 1	1	0.81 (0.55, 1.21)	0.66 (0.44, 0.98)	0.46 (0.31, 0.69)	<0.001	0.77 (0.66, 0.89)	<0.001
Model 2	1	0.90 (0.56, 1.45)	0.69 (0.44, 1.10)	0.50 (0.32, 0.80)	0.002	0.77 (0.65, 0.91)	0.003
Model 3	1	0.66 (0.38, 1.13)	0.39 (0.20, 0.77)	0.23 (0.10, 0.51)	<0.001	0.46 (0.30, 0.71)	0.001

Model 1 = adjusted for age and sex.

Model 2 = adjusted for age, sex, BMI (kg/m^2^), hypertension (SBP ≥ 190 mmHg and/or DBP ≥ 90 mmHg), physical activity (MET-h/day), antihypertension drug use (yes/no), smoking status (yes/no), drinking status (yes/no), tea drinking (yes/no), and total energy intake (kcal/d).

Model 3 = fully adjusted for age, sex, BMI (kg/m^2^), hypertension (SBP ≥ 190 mmHg and/or DBP ≥ 90 mmHg), physical activity (MET-h/day), antihypertension drug use (yes/no), smoking status (yes/no), drinking status (yes/no), tea drinking (yes/no), total energy intake (kcal/d), and daily nutrients intake (g/d), including saturated fatty acids, total dietary fibers, and total dietary aromatic amino acids (AAAs).

*P* < 0.05 indicates significant difference.

We further identified the relationship between the risk of CVD in patients with type 2 diabetes and food-derived BCAA intake per S.D. categorized by nutrients (Table 7). A higher consumption of mushrooms would reduce the risk of developing CVD for patients with type 2 diabetes by 25% for each 1–S.D. increase (95% CI = 7%, 39%, *P* = 0.009), followed by 24% per 1–S.D. increase in fruit consumption (95% CI = 3%, 41%, *P* = 0.027) and 24% per 1–S.D. increase in dairy and dairy product consumption (95% CI = 9%, 36%, *P* = 0.002), as well as per 1–S.D. increase in fruit, dairy and dairy product, whole grains, starchy vegetables and eggs consumption, respectively.

**Table 7:** Odd ratios and corresponding 95% confidence intervals with the fully adjustment model according to BCAA intake categorized by food groups and the risk of CVD in patients with type 2 diabetes.

**Food groups (g BCAA/per S.D.)**	**β**	**SE**	**(OR, 95%CI)**	***P***
Whole grains	−0.248	0.108	0.78 (0.63, 0.97)	0.022
Refined grains	0.074	0.090	1.08 (0.90, 1.29)	0.408
Soy and soy products	−0.266	0.217	0.77 (0.50, 1.17)	0.220
Non-starchy vegetables	−0.178	0.093	0.84 (0.70, 1.00)	0.055
Starchy vegetables	−0.240	0.094	0.79 (0.66, 0.95)	0.011
Legumes	−0.065	0.094	0.94 (0.78, 1.13)	0.490
Beans	0.186	0.150	1.20 (0.90, 1.62)	0.214
Tubers	0.116	0.091	1.12 (0.94, 1.34)	0.201
Mushrooms	−0.283	0.109	0.75 (0.61, 0.93)	0.009
Fruit	−0.277	0.126	0.76 (0.59, 0.97)	0.027
Eggs	−0.193	0.087	0.82 (0.69, 0.98)	0.027
Dairy and dairy products	−0.276	0.091	0.76 (0.64, 0.91)	0.002
Poultry	−0.075	0.082	0.93 (0.79, 1.09)	0.362
Processed meat	−0.010	0.086	0.99 (0.84, 1.17)	0.908
Red meat	0.017	0.103	1.02 (0.83, 1.24)	0.871
Fish and seafood	0.139	0.094	1.15 (0.96, 1.38)	0.137

Fully adjusted for age, sex, BMI (kg/m^2^), hypertension (SBP ≥190 mmHg and/or DBP ≥90 mmHg), physical activity (MET-h/day), antihypertension drug use (yes/no), smoking status (yes/no), drinking status (yes/no), tea drinking (yes/no), total energy intake (kcal/d), and daily nutrients intake (g/d) including saturated fatty acids, total dietary fibers, and total dietary aromatic amino acids (AAAs).

*P* < 0.05 indicates significant difference.

## Discussion

Our case–control study showed inverse associations between total dietary BCAA intake and CVD risk among patients with type 2 diabetes. Similarly, the highest quartiles of dietary isoleucine, leucine, and valine intake might provide beneficial effects against CVD among patients with type 2 diabetes when compared to the reference quartile. When the sources of food-derived dietary BCAA were taken into account, the levels of dietary BCAA among cases mainly came from animal-based foods rather than plant-based foods. Moreover, subjects with type 2 diabetes who regularly consumed whole grains, starchy vegetables, mushrooms, fruit, eggs, and dairy and dairy products had significantly lower risks of developing CVD.

Although the association between dietary BCAA and CVD risk among individuals with type 2 diabetes has not been reported, several studies have demonstrated the effect of dietary BCAA on metabolic biomarkers (e.g., TG, SBP, insulin, and HOMA-IR), obesity, type 2 diabetes, and CVD risk or mortality (Supplementary Tables 1, 2). A cross-sectional study of female twins in 1997 found that dietary BCAA was significantly associated with lower blood pressure (27). Likewise, US adults who consumed high amounts of essential amino acids were found to have lower all-cause and CVD mortality (28). This inverse relationship may be attributed to the protective effects of some specific amino acids of BCCA because total dietary BCCA is composed of amino acids that have some common features in their chemical structures and catabolism (29). In particular, a lower risk of CVD was associated with a higher intake of leucine (30), which was consistent with our study. A similar finding was found regarding the risk of obesity, and obesity risk was found to be reduced by 41.6, 37.4, and 44.1% as dietary isoleucine, leucine, and valine intake increased, respectively (31). Dietary isoleucine was positively associated with levels of fasting plasma glucose, triglycerides, and blood pressure, while dietary leucine was found to substantially decrease triglyceride levels (32). In addition, the negative association between CVD risk and dietary BCAA may likely be related to a low risk of atherosclerosis. The association between dietary BCCA, particularly leucine, and improvements in dyslipidemia and atherosclerosis-related factor development was recently found in subjects with higher serum triglyceride concentration (33). Dietary leucine and other amino acids prevented atherosclerosis by downregulating triglyceride-rich VLDL and inhibiting triglyceride biosynthesis in macrophages (29). Furthermore, each 1–S.D. increment of log 10-transformed dietary isoleucine, leucine, or valine intake was associated with a decrease in the risk of CVD mortality (33). This was also in line with our study, in which isoleucine was found to have no significant association with the risk of CVD. In contrast, the incidence of type 2 diabetes, CVD, and hypertension increased with dietary BCCA (34–36). Moreover, an increase in thrombosis formation occurs with increased ingestion of dietary BCAA (10). A similar finding from a meta-analysis showed that the restriction of dietary BCAA intake, which generally occurs with a simultaneous decrease in total protein intake, was inversely associated with impaired glucose in rodents (37). By reducing either dietary isoleucine or valine, hepatic insulin sensitivity was promoted, resulting in an improvement in glucose tolerance in rodents (11). In addition, a 2.46 unit increase in BMI was observed with the increased consumption of dietary isoleucine (11).

Dietary sources have been linked to the incidence of many chronic diseases (38–41). We hypothesized that plant-based BCAA was responsible for lowering CVD risk in our study. A higher incidence of CVD cases or CVD mortality was found in individuals who had a higher animal-to-plant protein ratio intake (40, 42). In addition, a higher risk of type 2 diabetes corresponds to greater levels of animal protein intake (41). While some studies found a null association between CVD risk and the consumption of fish, eggs, dairy, or plant protein (42), we found a reduction in risk with the increased intake of plant-based diets, eggs, and dairy and dairy products. An umbrella review of observational studies recently proposed convincing evidence of total dairy consumption in reducing the risk of hypertension, CVD, and diabetes (43). The elevation of HDL-c and PON-1, an enzyme that protects LDL from lipid peroxidation, was found after egg ingestions; therefore, individuals with metabolic syndrome who had consumed eggs presented a decrease in C-reactive protein levels, insulin levels, and insulin resistance, compared to baseline (44).

Dietary BCAA may drive the pathogenesis of cardio-metabolic diseases through the alteration of plasma BCAA concentrations since 80% of dietary BCAA consumption enters circulation (13). Studies have stated that increasing dietary BCCA may induce mTORC1 activity (14, 15) which is responsible for metabolic changes (45). Some believed that the elevation of serum BCAA levels is probably a consequence of disease rather than a cause (46) since endogenous factors, such as insulin, are able to regulate BCAA metabolism and degradation (47). In addition, the increase in amino acid oxidation was likely to be a consequence of impaired protein synthesis, which was caused by a limitation of diet-derived amino acid intake (48). Therefore, higher BCAA intake could decrease protein degradation and oxidative stress (49), while adequate BCAA consumption is essential for maintaining glucose homeostasis and body weight (9).

Our findings were in contrast with some other studies possibly due to the different amounts of dietary protein intake despite the sources of protein (e.g., egg and dairy). The associations of dietary BCAA or specific sourced proteins and the risk of diabetes or hypertension were found to have a non-linear relationship, indicating that the risk of diseases may increase as certain thresholds of some nutrients are surpassed (50, 51). Additional evidence for these dissimilarities of the risk of diseases might be related to genetic variations among individuals. Studies have demonstrated that a variety of individual genes are responsible for the difference in BCAA metabolic pathways and may affect the level of plasma BCAA together with dietary BCAA (52, 53).

## Limitations

There are some limitations that need to be taken into consideration when interpreting our results. First, we conducted a case–control study, so a causal relationship between exposures and diseases cannot be determined. Second, dietary information among participants was assessed by a food frequency questionnaire (FFQ); therefore, recall bias is inevitable. Third, selection bias still exists; however, we have tried to solve this bias by recruiting both groups from the same referral hospital. Fourth, although we considered some potential confounders in this study, we could not completely exclude other confounding factors that could interfere with these associations. Finally, we did not measure the levels of circulating BCAA which may also affect the reliability of the study.

## Recommendations

The results from this study have certain clinical and public health implications for the primary prevention of CVD, especially in individuals with type 2 diabetes. However, further prospective studies, including clinical trials, may be warranted to better understand the associations between dietary BCAAs and CVD risk among subjects with type 2 diabetes. Additional information on circulating BCAA should be collected, and if possible, genetic expression that is involved in the BCAA metabolic pathway may also be addressed to identify more details about the relationship between dietary BCAA and the risk of diseases.

## Conclusion

This case–control study indicated that the increased dietary intake of total BCAA and leucine might have some protective effect on the risk of CVDs in patients with type 2 diabetes. Plant-derived BCAA such as whole grains, mushrooms, and fruit, as well as eggs and dairy and dairy products, was associated with a lower incidence of CVD in individuals with type 2 diabetes.

## Data availability statement

The original contributions presented in the study are included in the article/Supplementary material, further inquiries can be directed to the corresponding authors.

## Ethics statement

The studies involving human participants were reviewed and approved by the Ethics Committee of the First Affiliated Hospital of Sun Yat-sen University [no. (2017)019]. The patients/participants provided their written informed consent to participate in this study.

## Author contributions

Y-bY and F-fZ contributed to the conception and design of the study and manuscript revision. LZ had responsibility for the data analyses and wrote the manuscript. JC, Y-hF, XS, S-yC, J-zL, W-lL, R-qO, J-rM, Y-jM, S-wZ, and K-yH collected the data and made a great contribution to the revised work. All authors have reviewed and approved the manuscript.

## Funding

This work was supported by grants from the Youth Program of the National Natural Science Foundation of China (Grant No. 81202197) and the Guangdong Natural Science Foundation (Grant No. 2015A030313011).

## Conflict of interest

The authors declare that the research was conducted in the absence of any commercial or financial relationships that could be construed as a potential conflict of interest.

## Publisher's note

All claims expressed in this article are solely those of the authors and do not necessarily represent those of their affiliated organizations, or those of the publisher, the editors and the reviewers. Any product that may be evaluated in this article, or claim that may be made by its manufacturer, is not guaranteed or endorsed by the publisher.

## Abbreviations

S.D: standard deviation
IQR: interquartile range
BCAA: branched-chain amino acids
CVD: cardiovascular diseases
BMI: body mass index
SBP: systolic blood pressure
DBP: diastolic blood pressure
WHR: waist–hip ratio
FPG: fasting plasma glucose
2hPG: 2-h postprandial blood glucose
HDL: high-density lipoprotein
LDL: low-density lipoprotein
TG: triglyceride
TC: total cholesterol
MET: metabolic equivalent
FFQ: food frequency questionnaire.
