# Supplementary material for: The association between dietary branched-chain amino acids and the risk of cardiovascular diseases in Chinese patients with type 2 diabetes: A hospital-based case–control study
Source: Front Nutr. 2022 Oct 14;9:999189. doi: 10.3389/fnut.2022.999189 (PMC9614346; doi:10.3389/fnut.2022.999189)
Supplement: Supplementary file 1 [file Data_Sheet_1.PDF]

Supplementary Material

Abbreviations: TLGS, the Tehran lipid and glucose study; HDNNCDS, the Harbin Cohort Study on Diet, Nutrition and Chronic Non-Communicable Diseases; NCHS, the National Center for Health Statistics; NHANES, the United States National Health and Nutritional Examination Survey; dietary BCAA, diet-derived branched-chain amino acids; Q1, the lowest quartile or quintile; Q4, the highest quartile; Q5, the highest quintile; T1, the lowest tertiles; T3, the highest tertiles; SD, standard deviation; CVD, cardiovascular diseases; HR, hazard ratio; OR, odd ratio; TG, triglyceride; FPG, fasting plasma glucose; SBP, systolic blood pressure; DBP, diastolic blood pressure; BMI, body mass index; β, regression coefficient; PCA, principle component analysis; AUC, area under the curve.

Supplementary Table 1 Summary the negative association between dietary BCAA and the risk of CVD or CVD related-factors

Authors	Year	Study design (Details)	Total study Participants	Age (Year)	Country	Type of amino acids	Type of comparison	Outcomes	Effect size (95% CI)	*P* value or *P* trend
Javidan, Sabour [1]	2015	Cross-sectional study	265 spinal cord patients	36.25 ± 10.76	Iran	Dietary leucine	Not reported	TG	β = -1.19	0.001
Jennings, MacGregor [2]	2016	Cross-sectional study	1 997 female twins	41.7 ± 12.1	UK	Dietary BCAA	Q5 vs. Q1	SBP	-2.3	0.01
								HOMA-IR	-0.1	0.02
								Insulin	-0.5	0.03
Mirmiran, Bahadoran [3]	2017	Population-based prospective cohort study (TLGS from 2006-2008, with a mean follow-up period of 6.7 years)	3 052	≥ 19	Iran	Dietary BCAA pattern	T3 vs. T1	CVD	HR = 1.00 (0.34-2.90)	0.92

(Continue) **Supplementary Table 1**

**Authors**	**Year**	**Study design (Details)**	**Total study Participants**	**Age (Year)**	**Country**	**Type of amino acids**	**Type of comparison**	**Outcomes**	**Effect size (95% CI)**	***P* value or *P* trend**
Mirmiran, Bahadoran [3] *(Continue)*	2017	Population-based prospective cohort study (TLGS from 2006-2008, with a mean follow-up period of 6.7 years)	3 052	≥ 19	Iran	Higher intake of arginine, cysteine, glutamic acid, glycine, histidine, leucine, tyrosine	T3 vs. T1	CVD risk	HR = 0.26 (0.09, 0.78)	0.03
Okekunle, Wu [4]	2018	Case-control study (from HDNNCDS population-based cohort study between 2010 and 2012	6 869 (3 009-normal weight and 1 109-obese only)	20-74	China	Total dietary BCAA	Q4 vs. Q1	Obesity risk	OR = 0.53 (0.35, 0.80)	0.003
						Dietary isoleucine	Q4 vs. Q1	Obesity risk	OR = 0.58 (0.39, 0.86)	0.005
						Dietary leucine	Q4 vs. Q1	Obesity risk	OR = 0.63 (0.44, 0.90)	0.007
						Dietary valine	Q4 vs. Q1	Obesity risk	OR = 0.56 (0.38, 0.82)	0.001

(Continue) **Supplementary Table 1**

**Authors**	**Year**	**Study design (Details)**	**Total study Participants**	**Age (Year)**	**Country**	**Type of amino acids**	**Type of comparison**	**Outcomes**	**Effect size (95% CI)**	***P* value or *P* trend**
Xu, Wang [5]	2021	Prospective cohort study (NCHS from 1988 to 1994, with 289,406 person-years of follow-up)	14 397	≥ 18	US	Total dietary BCAA	Per 1-S.D. increase	CVD mortality risk	HR = 0.85 (0.72, 0.99)	Not reported
						Dietary isoleucine	Per 1-S.D. increase	CVD mortality risk	HR = 0.81 (0.65, 1.00)	Not reported
						Dietary leucine	Per 1-S.D. increase	CVD mortality risk	HR = 0.83 (0.68, 1.00)	Not reported
						Dietary valine	Per 1-S.D. increase	CVD mortality risk	HR = 0.84 (0.71, 1.00)	Not reported

Supplementary Table 2 Summary the positive association between dietary BCAA and the risk of CVD or CVD related-factors

**Author**	**Year**	**Study design (Details)**	**Study Participants**	**Age (Year)**	**Country**	**Type of amino acids**	**Type of comparison**	**Outcomes**	**Effect size (95% CI)**	***P* value or *P* trend**
Javidan, Sabour [1]	2015	Cross-sectional study	265 spinal cord patients	36.25 ± 10.76	Iran	Dietary isoleucine	Not reported	FPG	$\beta = 0.69$	0.021
								SBP	$\beta = 1.17$	0.004
								DBP	$\beta = 1.19$	0.005
								TG	$\beta = 3.72$	<0.0001
Teymoori, Asghari [6]	2017	Population-based prospective cohort study (TLGS from 2008-2011, with a median follow-up period of 3.1 years)	6 493	20-70	Iran	Dietary BCAA pattern from PCA analysis	Q4 vs. Q1	Hypertension	OR = 1.83 (1.21-2.77)	0.002

(Continue) **Supplementary Table 2**

**Author**	**Year**	**Study design (Details)**	**Study Participants**	**Age (Year)**	**Country**	**Type of amino acids**	**Type of comparison**	**Outcomes**	**Effect size (95% CI)**	***P* value or *P* trend**
Okekunle, Wu [7]	2017	Case-control study (from HDNNCDS population-based cohort study between 2010 and 2012	8 824 (1804 cases of type 2 diabetes and 7 020 controls)	20-74	China	Total dietary BCAA	Q4 vs. Q1	Type 2 diabetes risk	OR = 1.56 (1.29, 1.89)	< 0.0001
						Dietary isoleucine	Q4 vs. Q1	Type 2 diabetes risk	OR = 1.55 (1.27, 1.89)	< 0.0001
						Dietary leucine	Q4 vs. Q1	Type 2 diabetes risk	OR = 1.66 (1.38, 1.99)	< 0.0001
						Dietary valine	Q4 vs. Q1	Type 2 diabetes risk	OR = 1.54 (1.27, 1.89)	< 0.0001
Teymoori, Asghari [8]	2019	Population-based prospective cohort study (TLGS from 2011-2014, with a median follow-up period of 3.1 years)	6 493	20-70	Iran	Dietary leucine / tryptophan	Q4 vs. Q1	Hypertension	OR = 1.22 (0.87, 1.72)	0.102
						Dietary leucine / threonine	Q4 vs. Q1	Hypertension	HR = 1.46 (1.01-2.12)	0.071
						Dietary (Leucine + Serine) / (Threonine + Tryptophan)	Q4 vs. Q1	Hypertension	HR = 1.48 (1.04-2.09)	0.026

(Continue) **Supplementary Table 2**

**Author**	**Year**	**Study design (Details)**	**Study Participants**	**Age (Year)**	**Country**	**Type of amino acids**	**Type of comparison**	**Outcomes**	**Effect size (95% CI)**	***P* value or *P* trend**
Okekunle, Zhang [9]	2019	Systematic review and meta-analysis	204 451	Not reported	Not reported	Total dietary BCAA	Q5 vs. Q1	Type 2 diabetes risk	Pooled OR = 1.58 (1.42, 1.76)	0.0003
			90 136			Dietary leucine	Q5 vs. Q1	Type 2 diabetes risk	Pooled OR = 1.43 (1.21, 1.70)	<0.00001
			90 136			Dietary isoleucine	Q5 vs. Q1	Type 2 diabetes risk	Pooled OR = 1.58 (1.42, 1.76)	<0.00001
			74 530			Dietary valine	Q5 vs. Q1	Type 2 diabetes risk	Pooled OR = 1.58 (1.42, 1.76)	0.01
			8 750			Dietary total BCAA	Q5 vs. Q1	Obesity risk	Pooled OR = 0.62 (0.47, 0.82)	0.0008

(Continue) **Supplementary Table 2**

**Author**	**Year**	**Study design (Details)**	**Study Participants**	**Age (Year)**	**State or Country**	**Type of amino acids**	**Type of comparison**	**Outcomes**	**Effect size (95% CI)**	***P* value or *P* trend**
Tharrey, Mariotti (10)	2020	Prospective cohort study	79 838	≥ 25	USA and Canada	Dietary BCAA pattern from PCA analysis	Q5 vs. Q1	CVD mortality risk	HR = 1.78 (1.24, 2.57)	<0.0001
Elovaris, Bitarafan (11)	2021	RCT (double-blinded)	15 healthy men	27 ± 3	Australia	Oral supplements contained either leucine (10 g), isoleucine (10 g), valine (10 g) or control	30 mins after administration	Plasma glucose	AUC_{t31-t1} = 137 ± 3 (Control)	0.098
									AUC_{t31-t1} = 135 ± 1 (Leucine)	
									AUC_{t31-t1} = 139 ± 2 (Isoleucine)	
									AUC_{t31-t1} = 143 ± 2 (Valine)	
								Plasma glucagon	AUC_{t31-t1} = 0 ± 87 (Control)	0.006
									AUC_{t31-t1} = 98 ± 73 (Leucine)	
									AUC_{t31-t1} = 231 ± 53 (Isoleucine)	
									AUC_{t31-t1} = 333 ± 104 (Valine)	

(Continue) **Supplementary Table 2**

**Author**	**Year**	**Study design (Details)**	**Study Participants**	**Age (Year)**	**Country**	**Main findings**
Xu, Jiang [12]	2020	Interventional controlled study	42 patients (21 type 2 diabetes and 21 healthy)	Not report	China	Dietary BCAA intake promoted platelets aggregation
Ha, Sakaki [13]	2021	Population-based prospective cohort study (NHANES from 1999-2010, follow-up until December 2015)	20 602	≥ 30	US	Higher intake of essential amino acids was associated with a lower CVD mortality risk
Yu, Richardson [14]	2021	Cross-sectional study	789	≥ 18	Not reported	Increased dietary isoleucine significantly gained more BMI by 2.46 units

References

1. Javidan AN, Sabour H, Nazari M, Soltani Z, Heshmat R, Larijani B, et al. Is the pattern of dietary amino acids intake associated with serum lipid profile and blood pressure among individuals with spinal cord injury? J Spinal Cord Med. 2017;40(2):201-12.

2. Jennings A, MacGregor A, Pallister T, Spector T, Cassidy A. Associations between branched chain amino acid intake and biomarkers of adiposity and cardiometabolic health independent of genetic factors: A twin study. International Journal of Cardiology. 2016;223:992-8.

3. Mirmiran P, Bahadoran Z, Ghasemi A, Azizi F. Contribution of dietary amino acids composition to incidence of cardiovascular outcomes: A prospective population-based study. Nutrition, Metabolism and Cardiovascular Diseases. 2017;27(7):633-41.

4. Okekunle AP, Wu X, Feng R, Li Y, Sun C. Higher intakes of energy-adjusted dietary amino acids are inversely associated with obesity risk. Amino Acids. 2018;51(3):373-82.

5. Xu B, Wang M, Pu L, Shu C, Li L, Han L. Association of dietary intake of branched-chain amino acids with long-term risks of CVD, cancer and all-cause mortality. Public Health Nutrition. 2021:1-11.

6. Teymoori F, Asghari G, Mirmiran P, Azizi F. Dietary amino acids and incidence of hypertension: A principle component analysis approach. Sci Rep. 2017;7(1):16838.

7. Okekunle AP, Wu X, Duan W, Feng R, Li Y, Sun C. Dietary Intakes of Branched-Chained Amino Acid and Risk for Type 2 Diabetes in Adults: The Harbin Cohort Study on Diet, Nutrition and Chronic Non-Communicable Diseases Study. Canadian Journal of Diabetes. 2017;42(5):484-92.e7.

8. Teymoori F, Asghari G, Farhadnejad H, Mirmiran P, Azizi F. Do dietary amino acid ratios predict risk of incident hypertension among adults? Int J Food Sci Nutr. 2019;70(4):387-95.

9. Okekunle AP, Zhang M, Wang Z, Onwuka JU, Wu X, Feng R, et al. Dietary branched-chain amino acids intake exhibited a different relationship with type 2 diabetes and obesity risk: a meta-analysis. Acta Diabetologica. 2019;56(2):187-95.

10. Tharrey M, Mariotti F, Mashchak A, Barbillon P, Delattre M, Huneau J-F, et al. Patterns of amino acids intake are strongly associated with cardiovascular mortality, independently of the sources of protein. International Journal of Epidemiology. 2020;49(1):312 - 21.

11. Elovaris RA, Bitarafan V, Agah S, Ullrich SS, Lange K, Horowitz M, et al. Comparative Effects of the Branched-Chain Amino Acids, Leucine, Isoleucine and Valine, on Gastric Emptying, Plasma Glucose, C-Peptide and Glucagon in Healthy Men. Nutrients. 2021;13(5).

12. Xu Y, Jiang H, Li L, Chen F, Liu Y, Zhou M, et al. Branched-Chain Amino Acid Catabolism Promotes Thrombosis Risk by Enhancing Tropomodulin-3 Propionylation in Platelets. Circulation. 2020;142(1):49-64.

13. Ha K, Sakaki JR, Chun OK. Nutrient Adequacy Is Associated with Reduced Mortality in US Adults. The Journal of Nutrition. 2021;151(10):3214-22.

14. Yu D, Richardson NE, Green CL, Spicer AB, Murphy ME, Flores V, et al. The adverse metabolic effects of branched-chain amino acids are mediated by isoleucine and valine. Cell Metab. 2021;33(5):905-22.e6.
